# Using isotopic fingerprints in gastropod shells to validate commercial production pathway and geographic provenance

**DOI:** 10.1098/rsos.231673

**Published:** 2024-05-15

**Authors:** Elise N. Boultby, Jasmin C. Martino, Ryan Baring, Zoë A. Doubleday

**Affiliations:** ^1^ College of Science & Engineering, Flinders University, Bedford Pk., Adelaide South Australia, Australia; ^2^ MARIS labs, Future Industries Institute, University of South Australia, Adelaide, South Australia, Australia

**Keywords:** provenance, seafood safety, abalone, stable isotopes, calcium carbonate, isotope ratio mass spectrometry

## Abstract

Growing demand for high-value seafood is fuelling provenance fraud, which threatens the sustainability of wild fisheries while posing biosecurity and human health risks. Here, we investigated carbon (*δ* ^13^C) and oxygen (*δ* ^18^O) isotopes in abalone shells (*Haliotis* sp.) to determine the production method and geographical provenance. Using X-ray diffraction and isotope ratio mass spectrometry, we found that shell mineralogy did not influence isotope values. Isotope values between wild and farmed sectors were statistically different, with 64% of individuals correctly classified as farmed or wild. Subsequently, we successfully distinguished the provenance of abalone collected from farms (with 83% of individuals correctly classified), as well as wild-caught abalone collected from four state jurisdictions (with 88% correctly classified). Carbon isotopes were strongly correlated to longitude, with both isotopes correlated to latitude. Overall, this study demonstrates the potential of isotopic fingerprints in gastropod shells to track the provenance of commercially valuable species.

## 1. Introduction

Seafood is one of the highest traded food commodities in the world, valued at an estimated 500 billion USD annually [1,2]. However, the growing global human population has put additional strain on seafood production, which nefarious players have exploited through fraudulent activities. Seafood fraud occurs when consumers or businesses are intentionally deceived about the species or provenance of a seafood product [3]. Seafood fraud can pose a health risk to consumers through the presence of allergens, bacteria, viruses and parasites that cannot be traced back to the source, as well as make it challenging to maintain sustainable harvesting practices [4,5]. As seafood is important to many different stakeholders, it is essential to ensure that any increase in production is following safe and sustainable practices. Provenance and traceability tools can directly address these challenges for seafood security and public safety by validating the origins of seafood and identifying fraudulent products.

To address the challenge of seafood fraud, there is increasing interest in the use of chemical profiling or fingerprinting to track the provenance of seafood species. To date, most seafood provenance studies have focused on developing isotopic and trace element profiles within soft tissues, or trace element profiles within calcium carbonate tissues [6]. However, isotopic profiles within calcium carbonate structures, such as shells, also show powerful potential for identifying region of origin as they can precisely record environmental information, such as water composition, salinity and temperature [7,8]. For example, stable isotope values of oxygen (*δ* ^18^O) in seawater, which are mediated by salinity, are primarily incorporated in equilibrium into carbonate structures with a temperature-related offset [9]. Furthermore, the universal expression of *δ* ^18^O values in carbonates across different marine species has demonstrated the efficacy of *δ* ^18^O values as a particularly useful provenance tool [10]. The stable isotope values of carbon (*δ* ^13^C) in carbonates are also largely in equilibrium with *δ* ^13^C of dissolved inorganic carbon of seawater, although a proportion is also related to diet and metabolic rates [11–13]. While *δ* ^18^O and *δ* ^13^C values in carbonates have the potential to be powerful tracers of provenance, most studies focus on palaeontological, fishery or ecological applications, with limited applications for provenance purposes [14].

Here, we investigated the potential of isotopic fingerprints (*δ* ^18^O and *δ* ^13^C values) within calcium carbonate shells to identify the provenance of a high-value gastropod species: abalone (*Haliotis* sp.). Abalone is particularly vulnerable to illegal fishing, with illegal catch estimated to make up more than 60% of total global catch [15,16]. Specifically, this research focuses on Australian abalone, a high-value product within the Australian seafood industry with an annual value of about 200 million AUD [17]. There are also suggestions that current management regulations and practices may be insufficient in controlling illegal trade occurring within the domestic Australian seafood industry [18,19]. Provenance techniques may assist authorities in identifying illegal catch of abalone, providing an avenue to determine the provenance of abalone across supply chains. While a handful of studies have analysed *δ* ^18^O values in abalone shells for ageing applications [20,21], the use of isotopes in provenance has yet to be explored. To assess the use of isotopic fingerprints for provenance applications we first investigated how the mineralogy of shells influenced isotope values, and, subsequently, assessed if *δ* ^18^O and *δ* ^13^C values in shells could distinguish between farmed and wild abalone or different catch locations of wild abalone across southern Australia. Lastly, we investigated geographic drivers of isotope variation in wild populations.

## 2. Material and methods

### 2.1. Shell collection and sample sites

**Figure 1.**
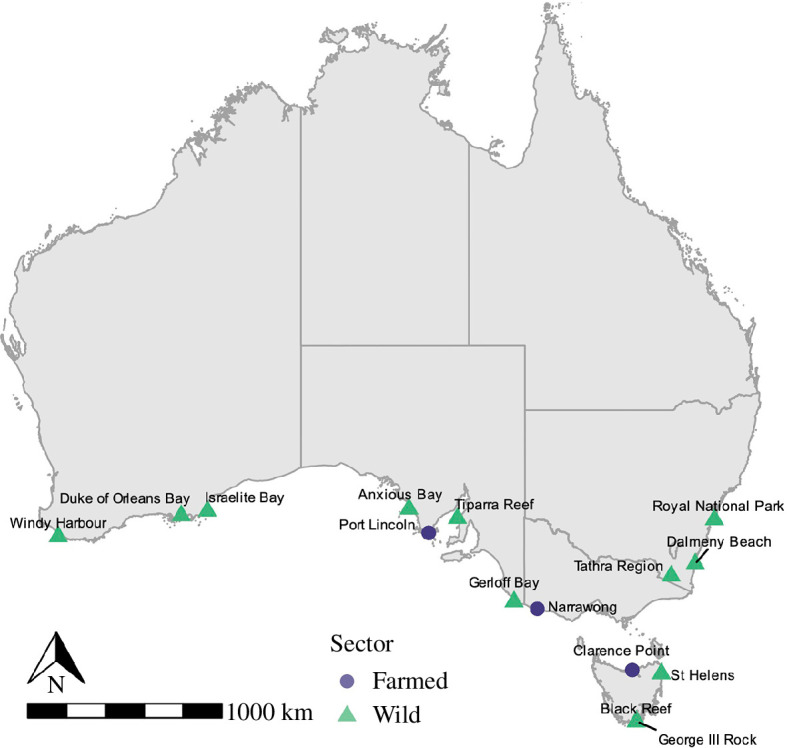
Map of Australia indicating sampling sites where farmed (purple circles) and wild (green triangles) abalone were collected.

The sample collection comprised the two main wild-caught species in Australia, blacklip abalone (*Haliotis rubra*) and greenlip abalone (*H. laevigata*); and the main farmed species in Australia, the jade tiger hybrid abalone (*H. laevigata* × *H. rubra*). Abalone shells were collected in collaboration with local industry and fishery scientists from 12 wild sites, as well as 3 farm sites, across Australia between September 2020 and January 2021 (figure 1 and table 1). Sample sites represented key abalone stocks in Southern Australia. Shells were freighted to the Future Industries Institute at the University of South Australia, where they were washed with 70% ethanol and length measured (mm) in preparation for processing.

**Table 1.** Information on collected samples of blacklip abalone (*H. rubra*), greenlip abalone (*H. laevigata*) and the jade tiger hybrid abalone (*H. laevigata* × *H. rubra*) collected from Australian state jurisdictions of New South Wales (NSW), South Australia (SA), Tasmania (TAS), Victoria (VIC) and Western Australia (WA).

sector and state	location	sample size	species
*Farmed*			
TAS	Clarence Point	10	*H. laevigata × H. rubra*
VIC	Narrawong	10	*H. laevigata × H. rubra*
SA	Port Lincoln	10	*H. laevigata × H. rubra*
*Wild*			
TAS	George III Rock	6	*H. rubra*
TAS	St Helens	10	*H. rubra*
TAS	Black Reef	10	*H. rubra*
NSW	Tathra Region	10	*H. rubra*
NSW	Dalmeny Beach	10	*H. rubra*
NSW	Royal National Park	10	*H. rubra*
SA	Anxious Bay	7	*H. laevigata*
SA	Gerloff Bay	10	*H. rubra*
SA	Tiparra Reef	10	*H. laevigata*
WA	Windy Harbour	10	*H. laevigata*
WA	Duke of Orleans Bay	10	*H. laevigata*
WA	Israelite Bay	9	*H. laevigata*

### 2.2. Influence of shell mineralogy on isotopic fingerprints

**Figure 2.**
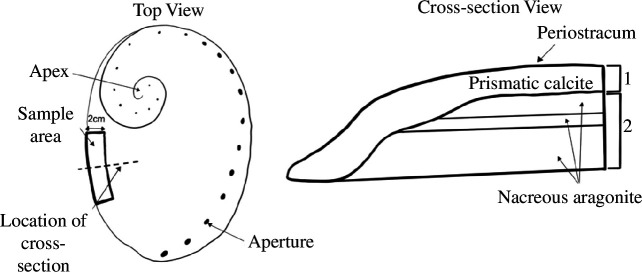
Schematic illustrating both top view and cross-section view of the sampling area of shell layers of abalone used to investigate the influence of shell mineralogy on isotopic fingerprints. To test the influence of shell mineralogy on isotopic fingerprints three samples were taken from each shell to separately assess the top layer (1), the remaining bottom layers (2) and all shell layers combined (1 + 2).

Abalone shells comprise calcium carbonate polymorphs, aragonite and calcite, with proportional contributions varying between species [22]. Abalone shells consist of an outer organic layer (periostracum), an intermediate prismatic layer of either aragonite or calcite and an internal nacreous layer, which is typically aragonite (figure 2) [23]. This mixture of shell mineralogy could influence *δ* ^18^O and *δ* ^13^C values within the shells, with aragonite typically being more enriched in *δ* ^18^O than calcite [24]. Therefore, to understand the effect of shell mineralogy on isotopic signatures, *δ* ^18^O and *δ* ^13^C values were analysed within the different shell layers of four farmed *H. laevigata × H. rubra* (jade tiger) individuals collected from Clarence Point, Tasmania. We selected farmed individuals to ensure relative consistency in shell thickness, size, age and environmental conditions, to better assess the influence of polymorph type on isotopic values.

Individual shells were rinsed with ethanol, with irregular material on the shell surface removed with a fine sanding drill attachment on a hand-held rotary tool. A 20 mm wide shell section was then removed from the terminal or growing edge of the shell (i.e. the newly formed portion of the shell) along the direction of growth. To remove the section, we used a fine, high-speed steel burr on a hand-held rotary tool (figure 2) [25]. A 20 mm shell section was selected as a conservative estimate of the annual growth rate of abalone to ensure that collected samples reflected the most recent environmental history or year of the individual [20,26]. Three samples were taken from each shell piece to separately assess the top layer, the bottom layers and all shell layers combined (*n* = 12) (figure 2). To protect the structural integrity of the shell, all shell sanding and grinding by the rotary tool was conducted at a constant, low speed to minimize heat effects on isotopic signatures [27]. Powder was ground further using an agate mortar and pestle (80 × 60 × 29 mm) and stored in vials until analysis.

To quantify the proportion of calcite to aragonite in each shell sample, samples were analysed using X-ray diffraction (XRD) at the University of Adelaide using a Bruker d8 Advance instrument. For each sample run, 800 μg of shell powder was placed onto a silicon wafer and a small spatula was used to ensure a flat, central layer. Four samples were run at a time and XRD data was then processed using TOPAS software and expressed as a percentage.

To understand whether *δ* ^18^O and *δ* ^13^C values in each shell sample varied due to calcite and aragonite composition, samples were then analysed using a GasPrep headspace and analysed by a connected Nu Horizon (Nu Instruments) isotope ratio mass spectrometer (IRMS) at the Mawson Analytical Spectrometry Services lab at the University of Adelaide. For each sample, 800 μg of shell powder (±100 μg) was weighed into exetainer vials using a 5 decimal place balance. The headspace was then purged for 2.5 min with helium gas. Each sample was injected with 100–120 μl of 103–104% phosphoric acid and held at 70℃ for 2 h, and the resulting CO_2_ gas was sampled and measured for its δ^13^C and δ^18^O composition. We measured samples against calcite standards (IAEA-CO-1 (CRM), IAEA-CO-8 (CRM), ANU-P3 and UAC), which were used to calculate external precision. Isotope values were reported relative to Vienna Pee Dee Belemnite and expressed in standard delta (δ) parts per thousand (‰),


$$\delta =\left(\frac{R_{sample}-R_{standard}}{R_{standard}}\right)\times 1000\left(‰\right),$$

where *R* is the ratio of ^13^C:^12^C or ^18^O:^16^O.

### 2.3. Provenance investigation: stable isotope analyses of wild and farmed abalone

Stemming from the sample optimization methods detailed above, a single 20 mm shell section was collected from all remaining abalone sample material that encompassed all shell layers. A total of 10 shells were sampled per region, except for George III Rock (6 samples), Anxious Bay (7 samples) and Israelite Bay (9 samples) (total samples = 142; table 1). Stable isotope analysis of *δ* ^18^O and *δ* ^13^C values was conducted using facilities at the University of Adelaide as described above. Duplicates were run every 10th sample to ensure instrument reliability. For all isotope analyses, external precision was better than ±0.08‰ for *δ* ^13^C and ±0.12‰ for *δ* ^18^O.

### 2.4. Statistical analyses

For the shell mineralogy component, univariate non-parametric PERMANOVA was conducted to investigate if the proportion of calcite, as well as isotope values, differed between layers. Non-transformed data were converted to Euclidean distance matrices with analyses performed using 10 000 permutations of the data. PERMANOVA was run with the shell layer as a fixed factor on percentages of calcite, *δ* ^18^O and *δ* ^13^C values in shells. Spearman’s rank correlations were conducted to identify significant relationships between *δ* ^18^O and *δ* ^13^C with calcite. Analyses were conducted only on calcite as combined percentages of calcite and aragonite equal 100% and therefore calcite analyses can be used to infer aragonite.

Subsequently, multivariate PERMANOVA was conducted to identify if there were significant differences in combined *δ* ^18^O and *δ* ^13^C values between farmed and wild sectors. PERMANOVA based on Bray–Curtis similarities was conducted using the production method as a fixed factor. Subsequently, wild and farmed sector data were analysed separately with PERMANOVA based on Bray–Curtis similarities for *δ* ^18^O and *δ* ^13^C combined (multivariate) and Euclidean distances for *δ* ^18^O and *δ* ^13^C separately (univariate) to identify if there were significant differences in isotopic signature between sites within the farmed and wild sectors. To identify differences specifically within the wild sector, PERMANOVAs were run with site as the fixed factor, where significant differences were detected, and *post hoc* pairwise *t*-tests were performed. For the *δ* ^18^O and *δ* ^13^C combined data only, separate canonical analysis of principal (CAP) coordinates were run with site or state as a factor to determine the specificity of isotopic signatures.

To determine the relationships between geographic location in wild-caught abalone and isotopic values, Spearman’s rank correlations were also conducted between both *δ* ^18^O and *δ* ^13^C values and the latitude and longitude of the sampling sites.

All PERMANOVA and CAP analyses were conducted in PRIMER/PERMANOVA+ (v. 7) software. Spearman’s rank correlations for the shell mineralogy were conducted in Origin Pro (2018). The spatial mapping and correlations between isotope values and geographic coordinates were conducted using R [28].

## 3. Results

### 3.1. Influence of shell mineralogy on isotopic fingerprints

Abalone shells were composed of a heterogeneous mix of calcite and aragonite, with some variability among individuals. There was a significant variation in aragonite and calcite composition between some layers (*p*‐value = 0.01). Per cent contribution in bottom layers was similar with 46% calcite and 54% aragonite, while a greater per cent contribution of aragonite was found in the combined layers (73%) and top layers (69%) (electronic supplementary material, figure S1). Although there was only a significant difference in calcite and aragonite percentages between samples from combined layers versus bottom layers (*p*‐value = 0.03), but not between combined layers and top layers, or bottom and top layers (*p*‐value = 0.4 and 0.06, respectively). There were differences in calcite percentage alone between layers (*p*‐value = 0.01), with combined layers containing 26.7% calcite, bottom layers containing 46.9% calcite and top layers containing 30.9% calcite. There was a variation between combined layers versus bottom layers (*p*‐value = 0.03) and not between combined layers and top layers, or bottom and top layers (*p*‐value = 0.41 and 0.06, respectively). Across shells, there was no significant difference in calcite and aragonite percentages within layers (*p*‐value = 0.35). The lack of statistical difference between the bottom and top layers indicates that there may be irregular layers of calcite and aragonite throughout the shell and that they could be challenging to separate out and analyse.

Both *δ* ^18^O and *δ* ^13^C values did not differ between shell layers (*p*‐value = 0.7). Spearman’s rank correlations between calcite-*δ* ^13^C and calcite-*δ* ^18^O were not significantly related (*p*‐value < 0.05). From these results, we concluded that sampling combined shell layers would be the most appropriate for provenance research because (i) no significant difference in *δ* ^18^O and *δ* ^13^C values was observed between shell layers and (ii) sampling combined shell layers is the most efficient and repeatable approach as we were unable to separate out layers with distinct mineralogy.

### 3.2. Provenance investigation: stable isotopes of farmed and wild abalone

Wild and farmed sectors were significantly different in *δ* ^18^O (*p*‐value = 0.0001) and *δ* ^13^C (*p*‐value = 0.03) values, with wild abalone more depleted in *δ* ^18^O and more enriched in *δ* ^13^C than farmed abalone. A CAP analysis between all farmed abalone and all wild-caught abalone correctly classified 64% of individuals as farmed or wild (electronic supplementary material, table S1). To better differentiate between harvest locations within each sector, all further statistical analyses were conducted separately for each sector.

#### 3.2.1. Farmed sector

**Figure 3.**
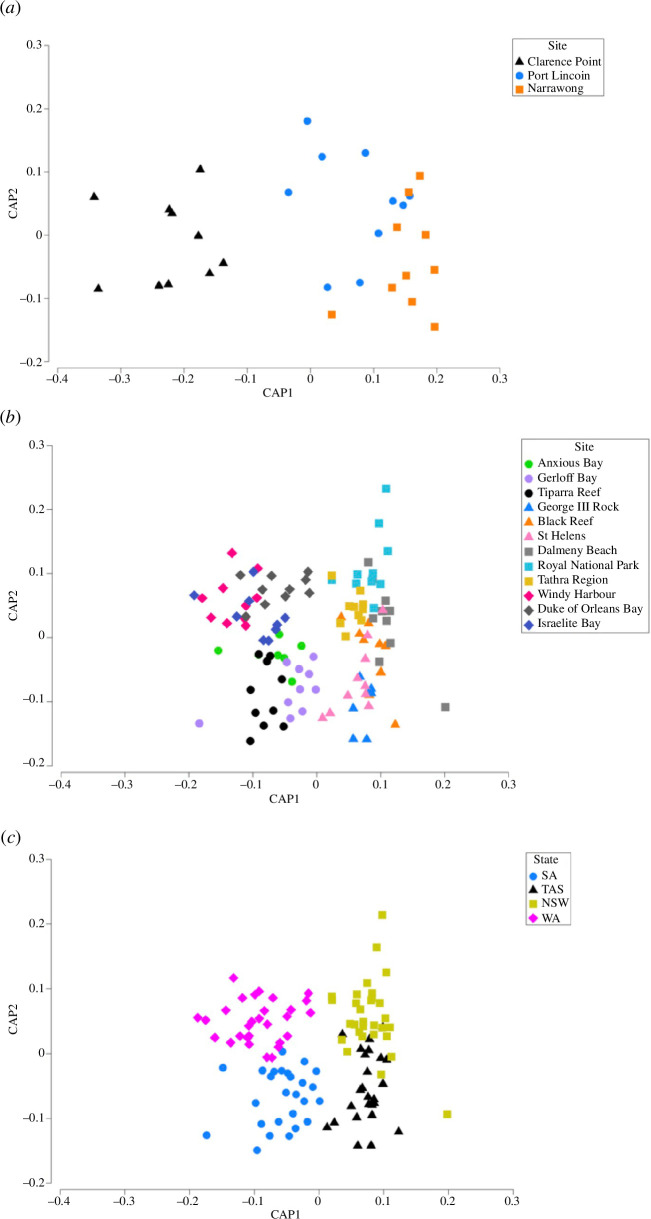
CAP using δ^13^C and δ^18^O values in shells comparing abalone collected from (*a*) farmed sites, (*b*) wild sites and (*c*) wild sites within each state jurisdiction.

*δ* ^13^C values in shells ranged from −1.38‰ in Victoria to −0.11‰ in South Australia, while *δ* ^18^O ranged from 0.87‰ to 2.72‰. Isotopic signatures were significantly different between sites in the farmed sector for *δ* ^18^O (*p*‐value = 0.0001), *δ* ^13^C (*p*‐value = 0.04), and *δ* ^18^O and *δ* ^13^C combined (*p*‐value = 0.0001). Pair wise tests comparing site for *δ* ^18^O and *δ* ^13^C showed that there was a significant difference between all site combinations (electronic supplementary material, table S2, *p*-values < 0.05). CAP analysis revealed that 83% of individuals were correctly classified back to the site, indicating that aquaculture sites were clearly separated by isotopic values (electronic supplementary material, table S3; figure 3*a*).

#### 3.2.2. Wild sector

**Figure 4.**
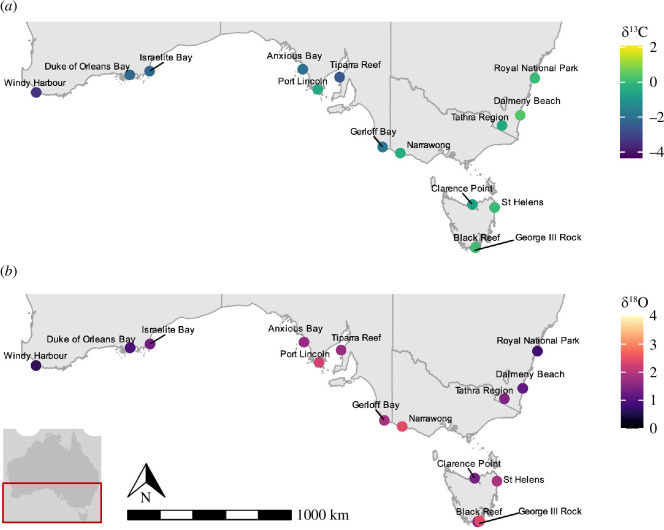
Map of southern Australia indicating the mean (*a*) δ^13^C and (*b*) δ^18^O values in shells of wild-caught abalone for each sampling site.

Mean *δ* ^13^C values in shells ranged from –3.35‰ in Windy Harbour to 0.45‰ in Dalmeny Beach, while mean *δ* ^18^O values ranged from 0.72‰ in Royal National Park to 2.33‰ in George III Rock (figure 4). Isotopic signatures were significantly different between sites in the wild sector with *δ* ^18^O, *δ* ^13^C, and *δ* ^18^O and *δ* ^13^C combined (*p*‐value = 0.0001). Pairwise tests indicated that 36 out of 55 site-to-site comparisons were significantly different between isotope values for *δ* ^18^O values (electronic supplementary material, table S4), while 37 out of 55 site-to-site comparisons were significantly different between *δ* ^13^C values (electronic supplementary material, table S5). However, when conducting pair wise tests on *δ* ^18^O and *δ* ^13^C combined, 48 out of 55 site-to-site comparisons were significantly different between isotope values.

**Figure 5.**
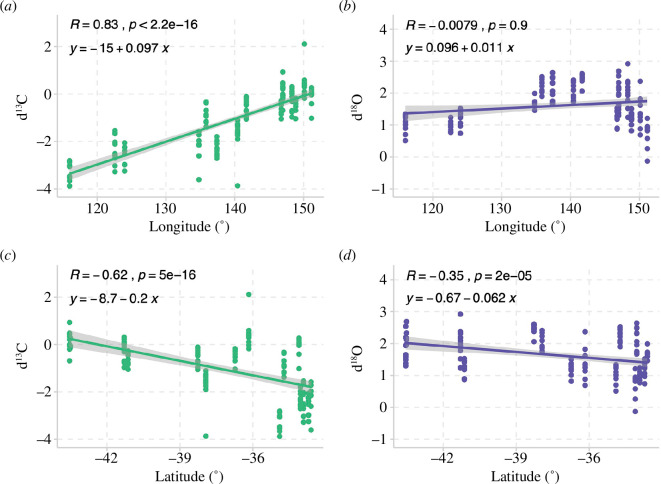
Correlation analysis between longitude (°) of sampling sites with (*a*) δ^13^C and (*b*) δ^18^O values in abalone shells, and latitude (°) of sampling sites with (*c*) δ^13^C and (*d*) δ^18^O values in abalone shells.

Correlation analyses were significant between *δ* ^13^C and both latitude and longitude, with a particularly strong relationship between *δ* ^13^C and longitude (*R* = 0.83; figure 5). In contrast, *δ* ^18^O values were only significant when correlated to latitude, and showed weaker relationships overall.

CAP analyses were conducted on *δ* ^18^O and *δ* ^13^C to assess isotopic separation between site locations. Correct classification of samples to the site based on CAP analysis found that 54% of samples were correctly classified back to the site (electronic supplementary material, table S6; figure 3*b*). However, in contrast, CAP analyses were also conducted to assess the isotopic separation and classification between Australian state locations, which were 88% correct, indicating a strong and reliable signature within each state location (electronic supplementary material, table S7; figure 3*c*).

## 4. Discussion

In this study, we investigated the influence of shell mineralogy on isotope signatures, as well as optimized the sampling method for analysing stable isotopes in abalone shells. While fractionation can differ between calcite and aragonite [29], importantly, we found that there was no significant difference in *δ* ^18^O and *δ* ^13^C values between shell layers, suggesting that the presence of calcite or aragonite in differing quantities within a sample would have relatively minimal influence on isotopic values. While our results were based solely on farmed individuals, which ensured relative consistency in shell thickness, size, age and environmental conditions among individuals, our results support field studies, which have also shown the minimal influence of shell mineralogy on *δ* ^18^O and *δ* ^13^C values in mollusc shells [24]. These results will have broad relevance to other gastropod and bivalve species with shells of mixed mineralogy, such as other abalone species, top shells, file shells, mussels and pen shells [22,30,31].

Isotopic fingerprints were statistically different between farmed and wild abalone, with moderate classification success (64%). This could be due to differing levels of temperature and salinity in farmed populations, which influences *δ* ^18^O and *δ* ^13^C in carbonates [8,9], as well as differences in diet, which can influence *δ* ^13^C in carbonates [13]. We also found that isotopic fingerprints could distinguish between some catch locations of wild abalone across southern Australia, with high classification success (88%) at the state level. As predicted, we found that latitude was correlated to *δ* ^18^O in abalone shells. This result aligns with the literature, where *δ* ^18^O in carbonates has been experimentally validated to reflect temperature [9], and thus reflect latitudinal temperature trends in the wild [8,10]. Carbon isotopes were also correlated to latitude and, interestingly, were strongly correlated with longitude and became increasingly positive in an easterly direction along the southern Australian coastline. This isotopic trend is probably due to the *δ* ^13^C values of the shells primarily reflecting the dissolved inorganic carbon of the seawater, although there may be a smaller proportion related to diet and metabolic rates [11–13]. Spatial variations in *δ* ^13^C values in the ocean also broadly align to temperature gradients, hence the correlation to latitude [32]. Ocean currents may be driving longitudinal trends in *δ* ^13^C values, with the Leeuwin Current flowing from west to east along the southern coast of Australia and influencing coastal environmental conditions (e.g. temperature but also salinity and nutrients) [33]. Also, at the time of sample collection, the 2020–2021 La Niña event was occurring (September 2020–March 2021), causing warm tropical water to flow down the Leeuwin Current, potentially influencing the western and southern Australian isotopic signatures [34]. However, isotopic signatures also showed some geographic inconsistencies; for instance, *δ* ^13^C values in Duke of Orleans Bay were distinct from Windy Harbour but more similar to Gerloff Bay. These inconsistencies may be driven by multiple, less prominent, interacting current systems (e.g. South Australian Current, Flinders Current and Tasman Outflow) [35]; however, further research focused on collecting more isotopic data from around Australia, alongside high-resolution salinity and temperature data around major current systems, is needed. Furthermore, we estimate that our shell sections roughly represented the last year of life of an individual based on a small study on blacklip abalone [20]. It is likely that our 20 mm sections did not precisely represent a year and that growth rates are likely to be variable among individuals, regions and species, which could influence isotopic values. While it would be possible to sample other regions of the shell if it is relevant to the question being answered (i.e. shell apex, which would represent the juvenile stage), it should be noted that shell sections cannot be precisely related to a period of time (i.e. calendar year).

Tracking the provenance of seafood presents promise in helping to combat seafood fraud and support sustainable fishing practices [6]. While other methods such as trace elements, fatty acids and DNA have been effectively used to identify provenance, these methods also have limitations. For instance, DNA markers reflect population structure and movement histories, rather than provenance *per se*, therefore, they are only useful when genetic differences exist among sampled populations [36]. The advantage of using shells or other biominerals is that chemical data are permanently locked within the hard structures and that they are easy to collect, store and archive for many years. This study has shown that *δ* ^18^O and *δ* ^13^C have the potential to be used as a provenance tool in abalone shells and other gastropod shells to distinguish between production methods, as well as different farms and wild populations. However, to produce a more precise signature, future research should consider combining *δ* ^18^O and *δ* ^13^C with other isotopic or elemental analyses, or even other biomarkers such as DNA [8,36,37].

As demand for high-value, ethical seafood grows, so does the importance of developing simple provenance methods that will facilitate sustainable practices while meeting production demands. This study showed that *δ* ^18^O and *δ* ^13^C fingerprints in shells can be used to distinguish between the origins of farmed and wild-caught abalone, based on relationships to water temperature and other environmental conditions. This study further demonstrated that shell mineralogy has a minimal impact on *δ* ^18^O and *δ* ^13^C values and thus provenance applications. To date, carbonate structures, such as shells, have been used surprisingly little in seafood provenance studies, despite their advantages over soft tissues [14,38]. Here, we demonstrate the potential of isotopic fingerprinting in gastropod shells for seafood provenancing applications which can support sustainable seafood practices into the future.

## Data Availability

Raw data is publicly available on Figshare and can be accessed online (https://doi.org/10.6084/m9.figshare.24494605). Precise geographic coordinates of collection locations remain confidential. However, approximate locations can be determined via the location names and map of sampling sites. Electronic supplementary material is available online [40].
